# Exploring the Association between Socioeconomic and Psychological Factors and Breastfeeding in the First Year of Life during the COVID-19 Pandemic in Thailand

**DOI:** 10.3390/ijerph20010130

**Published:** 2022-12-22

**Authors:** Sasitara Nuampa, Crystal L. Patil, Sudhathai Prasong, Kornkanok Kuesakul, Metpapha Sudphet

**Affiliations:** 1 Department of Obstetrics and Gynecological Nursing, Faculty of Nursing, Mahidol University, Bangkok 10700, Thailand; sudhathai.pra@mahidol.edu (S.P.); kornkanok.kue@mahidol.edu (K.K.); 2 Department of Human Development Nursing Science, College of Nursing, University of Illinois Chicago, Chicago, IL 60612, USA; cpatil@uic.edu; 3 Department of Obstetrics and Gynaecology, Siriraj Hospital, Mahidol University, Bangkok 10700, Thailand; metpaphasudphet@gmail.com

**Keywords:** breastfeeding duration, COVID-19, factor, psychology, socioeconomic, Thailand

## Abstract

Breastfeeding is essential for the survival, nutrition, and development of infants and young children. As a result of COVID-19’s effects of economic crises and psychological difficulties, breastfeeding outcomes have declined. The purpose of this study was to examine the association between socioeconomic and psychological factors with breastfeeding duration in the first year of life during the COVID-19 pandemic. Cross-sectional online surveys (*n* = 390) were conducted between August and November 2021. The participants were women aged 15 years and above who had given birth within 6–12 months before data collection and delivered in public hospitals in the top three provinces with the worst COVID-19 outbreaks during the second wave in Thailand. The average breastfeeding duration in this study was 6.20 months (±2.5) with a range of 1–12 months. Of mothers, 44.9% continued to breastfeed until between 6 and 12 months of age. In a multiple regression analysis, family income (Beta = 0.112, t = 1.988, *p* < 0.05), breastfeeding intention (beta = 0.097, t = 2.142, *p* < 0.05), intended breastfeeding duration (beta = 0.391, t = 8.355, *p* < 0.05), intention to receive vaccination (beta =0.129, t = 2.720, *p* < 0.05), and anxiety (beta = 0.118, t = 2.237, *p* < 0.05) were associated with breastfeeding duration in the first year of life (F (7, 382) = 20.977, *p* < 0.05, R^2^ = 0.278, R^2^ Adjusted = 0.264). During COVID-19, psychological factors were more strongly associated with breastfeeding duration in the first year of life than socioeconomic factors. Healthcare providers should promote breastfeeding intention, motivate COVID-19 vaccination intention, and support mental health among lactating mothers, particularly in the event of a pandemic.

## 1. Introduction

Breastfeeding (BF) is the cornerstone of the infant’s and young child’s survival, nutrition, development, and maternal health. Breast milk provides passive immune support through a complex of nutrients, immune cells, and other biologically active components that always support infant health but especially during a pandemic [1]. Despite concerns about transmission from the infected mother to the infant, global and national health stakeholders have unequivocally encouraged BF during the COVID-19 pandemic components that support infant health. Mothers should be counselled that the benefits of BF substantially outweigh the potential risks of infectious disease transmission [2,3]. Importantly, compiled global data indicated the presence of COVID-19 antibodies in breast milk following immunization [4].

The World Health Organization recommends exclusive breastfeeding for the first 6 months of life, followed by continued BF with appropriate complementary foods for up to 2 years and beyond [5]. The 2025 global nutrition target is to increase exclusive breastfeeding rates to 50% from the current rate of 41% [6]. The Thai National Statistics Office, in 2016 and 2019, reported exclusive breastfeeding rates at six months to be 23.1% and 14%, respectively, well below the international target of 50% [7].

Prior to the pandemic, socio-demographic factors associated with improved BF outcomes included higher education (OR 1.597, 95% CI 0.920, 2.775) and being multiparous (OR 2.035, 95% CI 1.202, 3.447) [8]. Maternal attributes positively associated with breastfeeding initiation and duration included higher maternal age [9,10,11], higher maternal education [12], and delayed return to work [13]. Furthermore, maternal psychological variables have also been shown to influence breastfeeding and can be seen as potential intervention targets. Studies have found that postpartum anxiety [14], BF intentions [15], and attitudes regarding BF [16] were predictive of BF duration. During the COVID-19 pandemic, a web-based study was performed in Ireland, Norway, Switzerland, the Netherlands, and the UK and reported the prevalence of major depressive symptoms and moderate to severe generalized anxiety symptoms were 13% and 10% of the breastfeeding women [17].

The COVID-19 pandemic and associated mitigation strategies, such as quarantines and social isolation negatively impacted household economics and increased psychological concerns [18]. The United Nations in Thailand reported that the socio-economic impact of COVID-19 in Thailand was affected by the loss of household income due to lay-offs, furloughs, or working hours loss, as well as disruption of the delivery of social services [19]. More women were feeling the high-risk impact of economic strain and mental health resulting from the COVID-19 pandemic [20]. Given the social distancing and other mitigating strategies along with hospital policies that separated mothers and newborns in the immediate postpartum [21], the impacts on maternal psychosocial health and on breastfeeding practice during the pandemic remain unclear [22,23]. Concerning BF and COVID vaccination attitudes, most breastfeeding women expressed a high level of unwillingness to receive COVID-19 vaccination due to a fear of harming their infants [24]. Some women reported a negative impact on BF and an allergic reaction following vaccination [25]. However, in this pandemic situation, little is known about the impact of socioeconomic and psychological factors on BF duration. The study investigated the following research question: “How did socioeconomic and psychological factors associate with BF in the first year of life during the COVID-19 outbreak?” The purpose of this study was to examine the associations of socioeconomic and psychological factors on BF duration in the first year of life during the COVID-19 pandemic among Thai postpartum mothers.

## 2. Methodology 

### 2.1. Setting

A cross-sectional online questionnaire was launched as a part of the project “Breastfeeding Behaviors and Experiences among Postpartum Mothers Encountering the Pandemic Situation of Coronavirus 2019: A Multi-Methods Approach”, which was conducted between August and November 2021 at three government hospitals in three provinces with high rates of COVID-19 related infection during the second wave in Thailand.

### 2.2. Sample

A sample size of 390 mothers was calculated by using the predominant breastfeeding proportion at six months in Thailand of 0.41 [7] with a 95% confidence interval and precision (d) of 0.05 and a 5% attrition rate [26]. The samples of this study were recruited by using a convenience sampling technique with eligible Thai women who had given birth within 6–12 months before the survey distribution, gestational age ≥ 37 weeks at childbirth, maternal age of 15 years old and above, and no history of COVID-19 infection. We identified potential participants from postpartum medical records and then contacted them by phone to explain the study and invite them to participate. Before being surveyed, women who agreed to participate signed electronic informed consent forms.

### 2.3. Measurement

The cross-sectional survey comprised closed-end questions via an online form, including data on demographics, pregnancy and childbirth information, an infant-feeding information form and four questionnaires (Fear of COVID-19 Scale, Attitude to COVID-19, State Anxiety Inventory Form Y-1, COVID-19 Vaccination Acceptability Tools). All 81-item questionnaires were distributed via an online link through a smartphone.

#### 2.3.1. The Demographics, Pregnancy and Childbirth Information Form

The 18-item questionnaire that was designed by researchers following the related literature. It aimed to survey the participants’ socio-demographic characteristics, such as maternal age (There are three age groups: the adolescent period [15–19 years old], the appropriate reproductive period [20–34 years old], and the late reproductive period [35 years old].) [27]; educational status; monthly family income; income status; work status; and family characteristics; the pregnancy and childbirth data included parity, pregnancy planning, maternal leave, maternal complications, and mode of delivery.

#### 2.3.2. Infant-Feeding Information Form

The 6-item questionnaire was designed by researchers following the related literature. It aimed to determine infant-feeding information including BF intention, previous BF experience, people influencing on BF practices, infant-feeding pattern and reasons to stop BF. For breastfeeding duration, the participants could feed human milk to their infants continuously from birth and might feed any water, liquid, food, or formula milk as well. Participants were asked how old their infant was when they stopped breastfeeding to define breastfeeding duration [28].

#### 2.3.3. Fear of COVID-19 Scale

The Fear of COVID-19 scale was used to assess postpartum mothers’ fear of COVID-19. This questionnaire has been shown to be both reliable and valid [29,30], with 7 items assessed on 5-point Likert scales ranging from 1 (strongly disagree) to 5 (strongly agree). The total fear score ranged from 7 to 35, with higher levels suggesting a strong fear of COVID-19 [29]. In our sample, the Cronbach’s alpha was 0.83.

#### 2.3.4. COVID-19 Vaccination Acceptability Tools

The COVID-19 vaccination attitude and intention were measured among postpartum mothers using the COVID-19 Vaccination Acceptability Tools [31], which included the first part of 28 items about a possible COVID-19 vaccination (*n* = 24), with researchers adding 3 items related to breastfeeding mothers (*n* = 3) and the second part for vaccination intention (*n* = 1). The first part assessed vaccination attitude with theoretical constructs, including perceived susceptibility to COVID-19, severity of COVID-19, benefits of a COVID-19 vaccine, barriers to COVID-19 vaccination, ability to be vaccinated, subjective norms, behavioral control, anticipated regret, knowledge, trust in the government, and BF related COVID-19. The perception statements were assessed on an 11-point scale (0–10) ranging from “strongly disagree” to “strongly agree.” This questionnaire has been shown to be reliable and valid [31]. The total sum of each question’s points resulted in vaccination attitudes having a minimum score of 0 and a maximum score of 270, with higher scores indicating a more positive attitude. The second part was 1 item of vaccination intention, an 11-point scale (0–10) from “very unlikely” to “very likely”, ranging 0–10, with higher scores denoting higher intention for COVID-19 vaccination [31]. In our sample, Cronbach’s alpha was 0.82. 

#### 2.3.5. State Anxiety Inventory Form Y-1

The State Anxiety Inventory Form Y-1 was used to assess maternal anxiety during COVID-19 (STAI-S) [32]. State anxiety refers to a one-time period or circumstance. Depending on the context, it is a limited or temporary state that is considered variable over time [33]. STAI-S consists of 20 items with four answer possibilities ranging from one to four points. To categorize and analyze the responses, scores ranging from 1 (almost never) to 4 (almost always) were assigned. The cumulative sum of the points for each question resulted in a minimum score of 20 and a maximum score of 80, with a higher score indicating a high level of anxiety. This questionnaire has been shown to be both reliable and valid [33], with a Cronbach’s alpha of 0.93 in our study.

#### 2.3.6. Attitude to COVID-19

The section of the Knowledge, Attitudes, and Practice (KAP) toward COVID-19 was used to assess attitudes toward the COVID-19 pandemic [34]. Attitudes toward COVID-19 were examined using two questions about agreement on COVID-19’s final control and confidence in winning the battle against COVID-19. The two questions were “(1) agree that the COVID-19 problem will be controlled”, with three responses (agree, disagree, and unknown), and “(2) confidence that Thai will successfully fight COVID-19”, with two answer options (confident and unconfident).

### 2.4. Data Collection

The researchers recruited participants who met the inclusion criteria through the medical records in postpartum units. Eligible women received telephone contact from researchers after they provided permission. All women received an explanation of the study procedures, and those who agreed to participate signed an electronic informed consent form sent to their smartphones. The women were sent links to an online self-administered questionnaire for the LINE application on their smartphones and asked to complete it within 24 h. Data collection was conducted from August to November 2021. According to ethical protocol, the only person who stored all data in digital files with confidential login details was the principal investigator.

### 2.5. Data Analysis

Descriptive analyses were presented as mean and standard deviation for normally distributed variables. For intergroup comparisons, the Mann–Whitney U-test was used for non-normally distributed variables for continuous variables. Chi-square tests were used to compare categorical variables between the groups. Multiple linear regression analysis was used to control for other factors that may affect BF duration for the first year of life. Socio-economic factors (employed status, educational level, financial status, and family income) and psychological factors (BF intention, Intended BF duration, Intention to COVID-19 vaccinate, attitude to COVID-19, attitude toward COVID-19 vaccination, anxiety, and fear toward COVID-19) were analyzed as independent variables in regression analysis. An overall 0.05 type-I error level was used to infer statistical significance.

## 3. Results

### 3.1. Demographic and Breastfeeding Data

The average age of the participants was 30.59 years (SD = 5.69). The average family income was USD 947.29 per month (SD = 1092). 59.7% (*n* = 233) of participants stay with the nuclear family. Almost half of the participants had their first child (*n* = 184, 47.2%). There were 83.4% (*n* = 325) of participants who were healthy mothers, while the rest had complications during pregnancy and the postpartum period. The majority of participants had caesarean sections (*n* = 213, 54.6%). For infants, the average age was 7.86 months (SD = 1.50) with a range of 6–12 months. Almost all babies (*n* = 348, 89.2%) had normal birth weights, with a mean birth weight of 3,070.50 g (SD = 423.81) and healthy babies (*n* = 382, 97.9%).

Regarding BF information, almost all of the participants in this study intended to breastfeed (*n* = 372, 95.4%). Nearly half of the participants had experience of BF with their previous infants (*n* = 164, 42.1%). The average BF duration was 6.20 months (±2.5) with a range of 1–12 months. Of mothers, 44.9% continued to breastfeed until between 6 and 12 months of age. In addition, participants who stopped BF provided several reasons during COVID-19; the top two reasons were insufficient breast milk supply (*n* = 178, 49.2%) and return to study/work (*n* = 155, 42.8%). 

### 3.2. Comparison of the Breastfeeding Duration in the First Year of Life According to the Socioeconomic Characteristics and Psychological Factors of Mothers during the COVID-19

Table 1 presents data on socioeconomic and psychological characteristics related to BF duration during the first year. A significant relationship was found between socioeconomic factors and BF duration during the first year. Marital status (χ^2^ = 17.079; *p* < 0.0001), family income (χ^2^ = 19.036; *p* < 0.0001), and educational status (χ^2^ = 10.380; *p* = 0.016) were positively associated with BF duration during the first year, whereas family income status (χ^2^ = −3.326; *p* = 0.001) was negative associated with BF duration during the first year. Significant psychological factors associated with BF duration during the first year were BF intention (χ^2^ = 27.490; *p* < 0.0001), intended BF duration (χ^2^ = 138.926; *p* < 0.0001), and intention to vaccinate (χ^2^ = 9.592; *p* = 0.008). Maternal age, work characteristics, attitude to COVID-19, fear of COVID, anxiety, and attitude toward vaccination were not associated with BF duration during the first year (Table 1).

### 3.3. The Predictive Factors on Breastfeeding Duration in the First Year of Life during COVID-19 Pandemic

Table 2 presents socioeconomic (family income) and psychological factors (BF intention, intended BF duration, intention to vaccination, and anxiety) associated with BF duration in the first year during the COVID-19 pandemic. In the regression analysis, increasing family income was more likely to continue BF during the first year of life (Beta = 0.112, t = 1.988, *p* < 0.05). Regarding psychological factors, BF intention (Beta = 0.097, t = 2.142, *p* < 0.05), intended BF duration (Beta = 0.391, t = 8.355, *p* < 0.05), intention to vaccination (Beta = 0.129, t = 2.720, *p* < 0.05), and anxiety (Beta = 0.118, t = 2.237, *p* < 0.05) were more likely to continue BF during the first year of life. The multiple linear regression model explained 27.8% (adjust R^2^ = 26.4%) of BF duration during the first year of life variance.

## 4. Discussion

This present study sought to examine whether socioeconomic and psychological factors influenced BF duration in the first year of life during the COVID-19 pandemic. The results revealed that family income, BF intention, intended BF duration, intention to vaccination, and anxiety were associated with BF duration. Interestingly, psychological factors are associated with a greater likelihood of BF duration than socioeconomic factors, particularly the duration of BF intention of mothers during COVID-19.

In previous studies, maternal intention was found to be an important predictor of BF duration [15,35]. Remarkably, this study showed BF intention greater than 6 months was the highest influence on BF duration during the first year of life. According to the theory of planned behavior (TPB), individual behavior is driven by behavioral intentions, where behavioral intentions are a function of three determinants, including an individual’s attitude toward behavior, subjective norms, and perceived behavioral control [36]. Breastfeeding intention might be a result of a positive attitude toward BF and social norms in a pandemic situation. Breastfeeding is accepted as not only the optimum source of nutrients for the infant but also as the protective shield for infants against infection through natural and passive immunity from mothers [2]. Mothers might receive positive BF messages from government information and promotion campaigns of breastfeeding benefits during COVID-19, which increased BF intention and outcomes [37,38] align with international recommendations [3]. Likewise, the previous study in Malaysia showed a higher percentage of mothers had a positive attitude towards BF during COVID [39]. Pandemic situations provided an opportunity to increase BF intention by helping parents to realize the benefits of BF for their infants, thereby ensuring the safety of the vulnerable infants.

According to this study, women who were more willing or intending to receive the COVID-19 vaccination were more likely to continue BF during the first years of life. However, the majority of the women in this study thought it was extremely unlikely that they would receive the COVID-19 vaccination (*n* = 308, 79%), with a significantly shorter BF duration compared to those who intended to receive vaccination (χ^2^ = 9.592; *p* = 0.008). Existing evidence suggests that maternal age, educational level, and public health message all influence acceptance of COVID-19 vaccination in BF mothers [40,41]. A previous study in Switzerland reported factors associated with SARS-CoV-2 vaccine acceptance in BF mothers that included an age older than 40 years and a higher educational level [40]. Moreover, positive information and social messages influenced the safety perception of the COVID-19 vaccination [41]. Thus, information about the effectiveness of COVID-19 vaccination, in particular in BF mothers, should be promoted so as to increase vaccine acceptance and improve BF duration in the first year of life.

Regarding anxiety during the COVID-19 pandemic, this study presented different results than previous studies. We found a positive relationship between the anxiety score and BF duration. It might occur as a result of how mothers can protect and safely infuse infant health with COVID-19 infection. Breastmilk could be a solution with a significant protective nutrient for their infant’s health. This is a key public health message to motivate and ensure BF values during the COVID-19 pandemic. The existing evidence found that the prevalence of anxiety about infection among women in the early postpartum period was increasing during the lockdown. Moreover, the study of Bangladesh found that primiparous mothers had higher anxiety than mothers who had previously delivered babies among breastfeeding mothers during COVID-19 [42]. The average anxiety score in this study was 51.85, which was higher than the middle interval score, and nearly half of the mothers (47.2%) were first-time mothers. However, mothers’ anxiety about COVID infection might have negatively impacted the emotional wellbeing of women [17]. Breastfeeding mothers should be supported to reduce anxiety of infection through the right protective methods and hygiene and simultaneously encouraged to continue BF. 

In regard to socioeconomic factors, high incomes for mothers were associated with long-term BF duration compared to lower incomes. A study in the USA found families with lower incomes were also found to have lower odds of breastfeeding (OR = 1.21; *p* = 0.001) [43]. However, there was some evidence that still conflicted with ideas that might depend on social norms in each context. For example, family income had not been found to be associated with breastfeeding in Japan [44]. During the COVID-19 pandemic in Thailand, the main factor affecting poverty was the loss of jobs or a reduction in working hours. Since 72% of household income comes from jobs, losses or reductions in working hours meant those households had to survive on savings. Some women might help their families with an early return to work or by seeking a new job that affected breastfeeding duration. While breast milk pumping might be discouraged during this time due to the risk of contamination by the COVID-19 virus. According to the study, Luthuli et al. found that financial pressures forced many mothers to return to work earlier than planned, resulting in changes to infant feeding practices or stopping BF entirely [45]. This highlights the need to target these populations in BF promotion efforts and reflects the fact that low-income families experience a number of barriers to receiving BF support and have poor access to the health care system, particularly during the COVID-19 pandemic.

Regarding the implementation to promote and support BF during the COVID-19 pandemic, psychological factors presented an important influence on continuing breastfeeding duration, particularly the BF intention program. Mothers should be provided with the importance of BF information and safety methods for their infants’ health during virus outbreaks in order to increase the intention of BF duration. Furthermore, lactational mothers were asked for informatic online intervention and easy platform access for COVID-19 vaccination to reduce anxiety and promote a positive attitude toward BF and vaccination, which can influence the continued BF duration and generate a solid protective shield for infants feeding children equally.

## 5. Conclusions

In COVID-19, psychological factors were more strongly associated with breastfeeding duration in the first year of life during COVID-19 than socioeconomic factors. Healthcare providers should provide the importance of BF and vaccination information, recommend safety methods for infants’ health, and provide a supportive program. In particular, low-income families may continue BF duration during the COVID-19 outbreak and correct information and sufficient BF support through an easily accessible platform may prevent maternal anxiety and increase BF duration in the first year of life.

## 6. Strengths and Limitations

This study has potential limitations. Since this is a cross-sectional study, it is not possible to establish a causal relationship between the determinant factors and BF duration during the COVID-19 pandemic. In addition, some mothers who had early BF weaning might have found it difficult to recall BF experiences, which affected recall bias in administering self-reporting online questionnaires. However, this study has several remarkable strengths. The recruitment process of this survey study selected volunteer participants from medical records to ensure inclusion criteria and directly contacted them to ask permission to send the questionnaire links, affecting high responsive rates (94.4%) and allowing them to ask clarification questions through online chat to minimize threat to validity. Moreover, this study conducted a convenience sample of three hospitals with a high rate of COVID-19 infection cases that might enhance validity. 

## Figures and Tables

**Table 1:** Comparison of the breastfeeding duration in the first year of life according to the socioeconomic characteristics and psychological factors.

Title 1	N (%) (N = 390)	BF Duration (Mean ± SD)	Z/χ^2^	*p*-Value
**Socioeconomic characteristics**				
Maternal age (y)				
<20 20–34 ≥35	12 (3.10) 278 (71.30) 100 (25.60)	4.68 ± 2.51 6.17 ± 2.46 6.39 ± 2.58	3.906	0.142
Marital status				
Single Marriage Separate/Divorce	42 (10.80) 334 (85.60) 14 (3.60)	4.61 ± 2.48 6.36 ± 2.43 6.16 ± 2.85	17.079	<0.0001 *
Family income (1 USD =32 baths)				
<10,000 10,000–30,000 30,001–60,000 >60,000	66 (16.90) 207 (53.10) 78 (20.00) 39 (10.00)	5.56 ± 2.63 5.94 ± 2.38 6.48 ± 2.27 7.76 ± 2.72	19.036	<0.0001 *
Family income status				
Enough Not enough	230 (59.00) 160 (41.00)	6.53 ± 2.49 5.65 ± 2.43	−3.326	0.001 *
Educational status				
Primary school High school Bachelor degree ≥ Master degree	12 (3.10) 180 (46.20) 168 (43.10) 30 (7.70)	5.42 ± 2.91 5.79 ± 2.39 6.49 ± 2.51 6.95 ± 2.60	10.38	0.016 *
Work characters				
Housewife Inside work Outside work	128 (32.8) 82 (21.0) 180 (46.2)	6.50 ± 2.27 6.21 ± 2.60 5.92 ± 2.59	5.142	0.076
**Psychological characteristics**				
Breastfeeding intention				
Yes No Unsure	372 (95.4) 11 (2.8) 7(1.8)	6.32 ± 2.41 2.00 ± 1.10 4.71 ± 2.69	27.490	<0.0001 *
Intended BF duration (months)				
<6 6–12 13–24 >24	148 (37.9) 155 (39.7) 81 (20.8) 6 (1.5)	4.36 ± 1.93 7.28 ± 2.22 7.29 ± 2.20 6.83 ± 2.32	138.926	<0.0001 *
Attitude to COVID-19 ^1^				
Agree Disagree Unknown	210 (53.8) 79 (20.3) 101 (25.9)	6.31 ± 2.39 6.09 ± 2.48 5.93 ± 2.73	1.508	0.471
Attitude to COVID-19 ^2^				
Confident Unconfident	131 (33.60) 259 (66.40)	6.21 ± 2.39 6.15 ± 2.56	−0.093	0.926
Fear to COVID-19				
Low level High level	31(7.9) 359(92.1)	6.36 ± 2.35 6.15 ± 2.52	−0.211	0.833
Intention to vaccination				
Very unlikely Uncertain Very likely	308 (79.0) 71 (13.2) 11 (2.8)	4.29 ± 2.95 5.61 ± 2.41 6.36 ± 2.46	9.592	0.008 *
	**Min-Max**	**Score (Mean ± SD)**	***p***	***p*** **-value**
Anxiety	20–78	51.85 ± 10.60	0.131	0.009 *
Attitude toward vaccination	86–270	175.56 ± 32.49	−0.022	0.669

^1^ Attitude to COVID-19 question 1 is “Agree that the COVID-19 problem will be controlled”; ^2^ Attitude to COVID-19 question 2 is “Confidence that Thai will successfully fight COVID-19”; Data are shown as N (%) and mean ± standard deviation; Chi-squared testing was used for categorical variables; *p* is correlation coefficient; Z-score in Mann–Whitney U test for continuous variables with not normally distributed data; * *p*-value < 0.05.

**Table 2:** Socioeconomic and psychological factors affecting of breastfeeding duration in the first year of life (*n* = 390).

Factors	B	SE	Beta	T	*p*	95% CI
**Socioeconomic Factors**						
Employed status	−0.209	0.136	−0.073	−1.532	0.126	−0.476 to 0.059
Educational level	0.111	0.198	0.030	0.561	0.575	−0.279 to 0.501
Financial status	−0.305	0.263	−0.060	−1.158	0.248	−0.823 to 0.213
Family income (per month)	0.329	0.166	0.112	1.988	0.048 *	0.004 to 0.655
**Psychological Factors**						
BF intention	0.782	0.365	0.097	2.142	0.033 *	−1.500 to −0.064
Intended BF duration	0.129	0.015	0.391	8.355	<0.0001 *	0.099 to 0.160
Intention to COVID-19 vaccinate	0.145	0.053	0.129	2.720	0.007 *	0.040 to 0.250
Attitude toward COVID-19 vaccination	−0.007	0.004	−0.085	−1.746	0.082	−0.014 to 0.001
Attitude toward COVID-19 ^1^	−0.103	0.146	−0.035	−0.705	0.481	−0.390 to 0.184
Attitude toward COVID-19 ^2^	0.325	0.267	0.062	1.220	0.223	−0.199 to 0.849
Anxiety	0.028	0.012	0.118	2.237	0.026 *	0.003 to 0.052
Fear toward COVID-19	0.050	0.027	0.100	1.886	0.060	−0.002 to 0.103

^1^ Attitude to COVID-19 question 1 is “Agree that the COVID-19 problem will be controlled”; ^2^ Attitude to COVID-19 question 2 is “Confidence that Thai will successfully fight COVID-19”; R = 0.527, R^2^ = 0.278, Adjusted R^2^ = 0.264; B, beta coefficient; CI, confidence interval; SE, standard error; T, t-score of the regression model; * *p*-value < 0.05.

## Data Availability

Not applicable.
